# How Has the Pandemic Affected Women in Medicine? A Survey-Based Study on Perceptions of Personal and Career Impacts of COVID-19

**DOI:** 10.1089/whr.2021.0031

**Published:** 2021-09-20

**Authors:** Christina Brown, Shikha Jain, Lekshmi Santhosh

**Affiliations:** ^1^ Department of Medicine, Rush Medical College, Chicago, Illinois, USA.; ^2^ Department of Medicine, Hematology, Oncology and Cell Therapy, University of Illinois Cancer Center in Chicago, Chicago, USA.; ^3^ Department of Medicine, Pulmonary/Critical Care Medicine and Hospital Medicine, University of California, San Francisco, San Francisco, California, USA.

**Keywords:** COVID-19, pandemic, women physicians

## Abstract

***Background:*** Gender inequity is apparent for women in medicine. With the onset of the COVID-19 pandemic, there are concerns about how women physicians are personally and professionally impacted.

***Materials and Methods:*** Participants of the Women in Medicine Summit were anonymously surveyed about their perspectives about COVID-19 affecting women in medicine. Questions were centered around perceived gender bias, productivity expectations, and stressors.

***Results:*** At the Women in Medicine Summit, 454 attendees were invited to complete the survey with a response rate of 27% (*n* = 124). Of those participants, 46% of participants perceived gender bias in the workplace, with 39% citing further inequities with intersectional identities (*p* < 0.05). Productivity expectations were reported to be higher than before the pandemic in 41% of survey participants. The majority of respondents (70%) reported experiencing high levels of stress during the pandemic, compared with only 16% reporting high levels of stress before the pandemic (*p* < 0.05).

***Discussion:*** It is clear that women physicians are experiencing the pandemic differently. Gender bias is a common occurrence, especially by individuals with intersectional identities. These stressors are not new to women in medicine, but with the overriding impact of the pandemic, higher expectations for productivity, and increased personal responsibilities, employers should focus on preventing further exacerbations of gender inequity in medicine.

## Introduction

For women in academic medicine, gaps remain at the highest levels of leadership^1^; the economic impact of COVID-19 could further exacerbate these gaps.^2^ It is crucial to understand how women in medicine perceive the personal and professional impacts of COVID-19 on their lives.

## Materials and Methods

From October to December 2020, Women in Medicine Summit attendees were invited by e-mail to anonymously complete a survey about the impact of COVID-19 on their professional and personal lives using Qualtrics survey software. Multiple-choice and free-text questions asked about COVID-19's impact on perceived gender bias, expectations within and outside of work, and stress. These questions were developed based on literature review surrounding the impact of COVID-19 on women in medicine.^2^ The questions were tested through cognitive interviewing by other Women in Medicine Summit colleagues. Participants received monthly e-mail reminders for 3 months to complete the survey. One-sample Wilcoxon tests were performed to compare the median for all survey questions with the exception of comparing stress levels prepandemic and currently where two-sample Wilcoxon test was performed to compare the medians. Statistical analysis was performed by R software (version 4.0.3). Significance level was set at 0.05. Descriptive statistics and thematic analysis of comments were also performed. This study was considered exempt by the University of California-San Francisco institutional review board.

## Results

Demographics showed that the majority of survey respondents (98.5%) were female, from >11 specialties. Participants ranged from medical students to physicians in practice for >20 years. Participants identified their face as majority Caucasian 62.7%, followed by Asian (16.4%), and Latinx (7.5%) (Table 1). Almost half of the respondents (46%) reported perceiving gender bias to a “Moderate” or “Large” extent (*p* < 0.05). Women reported difficulties with career advancement, caregiving leave, and access to leadership opportunities. Respondents felt that gender bias was experienced even more frequently by women with intersectional identities, such as black, Latinx, and Lesbian Gay Bisexual Transexual Queer-identifying women, with 39% citing that bias occurred to a “Moderate” or “Large” extent (*p* < 0.05).

**Table 1.** Demographics of Participants in the Women in Medicine Summit Survey

Gender identity		
Male	0.00%	0
Female	98.48%	65
Nonbinary	0.00%	0
Prefer not to answer	1.52%	1
Racial/ethnic identity		
African American	5.97%	4
Asian	16.42%	11
Caucasian	62.69%	42
Latinx	7.46%	5
Native American	1.49%	1
Pacific Islander	0.00%	0
Other	4.48%	3
Prefer not to answer	1.49%	1
Specialty		
Medicine	26.56%	17
Surgery	1.56%	1
Pediatrics	3.13%	2
Neurology	6.25%	4
Psychiatry	4.69%	3
Anesthesiology	0.00%	0
Family medicine	7.81%	5
Medicine subspecialty	10.94%	7
Surgical subspecialty	7.81%	5
Radiology	3.13%	2
Pathology	0.00%	0
Other	28.13%	18
Currently hold leadership position		
Yes	56.92%	37
No	43.08%	28
Profession focus		
Clinical	36.76%	25
Research	7.35%	5
Administrative	8.82%	6
Clinical and research	23.53%	16
Clinical and administrative	14.71%	10
Other	8.82%	6
Years in practice		
Still in training (medical student, resident)	25.00%	17
1–3 years	10.29%	7
4–6 years	11.76%	8
7–10 years	7.35%	5
10–13 years	14.71%	10
14–16 years	4.41%	3
17–20 years	7.35%	5
20+ years	19.12%	13

Regarding productivity in the workplace, 41% reported perceiving higher expectations, whereas 8% reported fewer expectations compared with the prepandemic environment. In addition, 33.8% of respondents reported being “Worried” and “Very Worried” about their level of productivity in nonclinical work such as research and medical education. Thematic analysis of respondents' comments regarding productivity expectations were cited as unclear. Before the pandemic, 14% of participants reported feeling “Uncertain” and “Very Uncertain” about their careers. During the pandemic, this uncertainty in participants' professional future increased to 37%.

Whereas 16% of participants self-reported being “Stressed” or “Very Stressed” before the pandemic, during the pandemic 70% of participants reported feeling “Stressed” or “Very Stressed,” indicating stress levels are significantly different compared with prepandemic and current pandemic (*p* < 0.05).

**FIG. 1.**
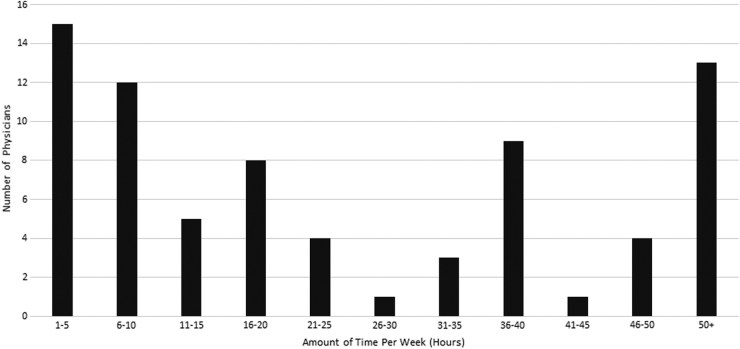
Self-reported number of hours per week by physicians that were spent on childcare, eldercare, and household tasks during the COVID-19 pandemic (*n* = 75).

Participants reported spending a wide range of hours on either childcare, eldercare, and/or household tasks (Fig. 1). Of participants who had children <10 years (*n* = 21), 71% reported that the pandemic had moderately or greatly limited access to opportunities to advance in their careers. Inconsistent childcare and remote learning were perceived as the biggest contributors to this phenomenon.

## Discussion

In this survey-based study of women in medicine, gender bias was perceived as common and stress levels were reported to be increased due to the pandemic. The stressors women in medicine face are not new, but the pandemic has wrought greater impacts on women's personal and professional lives.^3^ Almost half of physician mothers (41%) in one study met criteria for moderate or severe anxiety during the pandemic.^4^ Women have felt productivity expectations by institutions seemed unclear or even increased during the pandemic.

Even before the pandemic, women spent more time on family and household tasks: asking for accommodations was perceived as being less committed to medicine.^3^ The pandemic's impact on school/childcare has exacerbated work-life imbalance, greatly impacting women and those with intersectional identities.^5^ Research productivity has decreased in women, with fewer women first authors in COVID-19 publications,^3^ which could negatively impact their academic advancement and promotion trajectories.^5^

As this survey was completed during the Women in Medicine summit, an event focused on amplifying women in medicine and working toward gender equity. Selection bias could account for participants being more attuned to gender bias. This was a cross-sectional survey administered briefly during the pandemic; longitudinal surveys of larger samples would improve the results' validity. Survey responses relied on self-report, subjecting the study to recall bias as participants compared current perceptions with perceptions before the pandemic. Moreover, response bias could have impacted the results as those who were experiencing excessive stress could have been either more likely or less likely to respond to the survey. In addition, objective metrics of stress and gender bias were not conducted, so it is difficult to validate if participants could have had an optimistic perception of prepandemic stress and bias levels. In addition, demographic data obtained showed predominant participants were Caucasian women (62.7%) specializing in Internal Medicine and related subspecialties (26.6%) who were still in training (25.0%). This limitation prevents being able to generalize to all women in medicine, specifically those at mid-career. Survey responses were not collected from a matched male cohort to more explicitly compare how experiences and perceptions differ between women and men. The survey was open for 3 months, with a total of three e-mail reminders for participants to complete.

Women in medicine are juggling caregiving responsibilities and household tasks with perceived increased productivity expectations in the workplace, all amidst the consistent strain of gender bias and a pandemic. Institutions can prevent further attrition of women physicians in academic medicine by setting clear productivity expectations, assisting with grant funding deadline extensions, subsidizing caregiving responsibilities, adopting flexible work schedules, and promoting tools to formally document COVID-19-related career contributions.^2,6^

## Conclusion

The COVID-19 pandemic has impacted women physicians personally and professionally, and further research should quantify exactly how lectures, publications, and projects could have changed as a result. Women are perceiving higher stress levels, increased uncertainty in their careers, and increased demands upon household and family tasks. It is essential to intervene now to prevent further widening of the gender gap in medicine, using strategies such as encouraging flexible work schedules, concretely supporting childcare and eldercare needs, and encouraging structured tools to document in a standardized way of how individuals' academic productivity was impacted by the pandemic.^2^

## References

[1] AAMC. 2018–2019 The state of women in academic medicine: exploring pathways to equity. 2021. Available at: https://www.aamc.org/data-reports/data/2018-2019-state-women-academic-medicine-exploring-pathways-equity Accessed 13, 2021

[2] Woitowich NC, Jain S, Arora VM, Joffe H. COVID-19 threatens progress toward gender equity within academic medicine. Acad Med 2021;96:813–816 33003040 10.1097/ACM.0000000000003782 PMC7543905

[3] Spector ND, Overholser B. COVID-19 and the slide backward for women in academic medicine. JAMA Netw Open 2020;3:e2021061 32940676 10.1001/jamanetworkopen.2020.21061

[4] Linos E, Halley MC, Sarkar U, et al. Anxiety levels among physician mothers during the COVID-19 pandemic. Am J Psychiatry 2021;178:203–204 33517747 10.1176/appi.ajp.2020.20071014

[5] Woodhams C, Dacre J, Parnerkar I, Sharma M. Pay gaps in medicine and the impact of COVID-19 on doctors' careers. Lancet 2021;397:79–80 33338438 10.1016/S0140-6736(20)32671-4 PMC9753502

[6] Brubaker L. 2021. Women physicians and the COVID-19 pandemic. JAMA 2020;324:835–836 10.1001/jama.2020.14797 32735329

